# Vasculature-Associated Lymphoid Tissue: A Unique Tertiary Lymphoid Tissue Correlates With Renal Lesions in Lupus Nephritis Mouse Model

**DOI:** 10.3389/fimmu.2020.595672

**Published:** 2020-12-15

**Authors:** Md. Abdul Masum, Osamu Ichii, Yaser Hosny Ali Elewa, Yuki Otani, Takashi Namba, Yasuhiro Kon

**Affiliations:** ^1^ Laboratory of Anatomy, Department of Basic Veterinary Sciences, Faculty of Veterinary Medicine, Hokkaido University, Sapporo, Japan; ^2^ Department of Anatomy, Histology and Physiology, Faculty of Animal Science and Veterinary Medicine, Sher-e-Bangla Agricultural University, Dhaka, Bangladesh; ^3^ Laboratory of Agrobiomedical Science, Faculty of Agriculture, Hokkaido University, Sapporo, Japan; ^4^ Department of Histology, Faculty of Veterinary Medicine, Zagazig University, Zagazig, Egypt

**Keywords:** tertiary lymphoid tissue, lupus nephritis, chemokine, kidney, renal lesions, dexamethasone

## Abstract

Lupus nephritis (LN) is a common complication in young patients and the most predominant cause of glomerulonephritis. Infiltrating immune cells and presence of immunocomplexes in the kidney are hallmarks of LN, which is closely associated with renal lesions (RLs). However, their regulatory mechanism in the kidney remains unclear, which is valuable for prevention of RL development. Here, we show the development of vasculature-associated lymphoid tissue (VALT) in LN, which is related to renal inflammatory cytokines, indicating that VALT is a unique tertiary lymphoid tissue. Transcriptomic analysis revealed different chemokines and costimulatory molecules for VALT induction and organization. Vascular and perivascular structures showed lymphoid tissue organization through lymphorganogenic chemokine production. Transcriptional profile and intracellular interaction also demonstrated antigen presentation, lymphocyte activity, clonal expansion, follicular, and germinal center activity in VALT. Importantly, VALT size was correlated with infiltrating immune cells in kidney and RLs, indicating its direct correlation with the development of RLs. In addition, dexamethasone administration reduced VALT size. Therefore, inhibition of VALT formation would be a novel therapeutic strategy against LN.

## Introduction

The vital functions of the mammalian kidney include filtration of blood to excrete metabolic wastes and toxins through urine, maintenance of body fluid balance, pH, and absorption of minerals to sustain life (1). The prevalence of patients suffering from chronic kidney disease (CKD) is 8–13%, overall (2). Moreover, it has been estimated that more than 19 million people in the USA and about 22% of adult humans in Japan suffer from complications from CKD (3, 4). Therefore, CKD is a major public health concern as is associated with end-stage renal disease and cardiovascular complications (5). Since kidney is a non-regenerative organ, understanding the pathogenesis of CKD and identification of therapeutic targets is valuable for developing remedial strategies. Different studies using human biopsy samples and experimental animals showed that infiltration of inflammatory cells in the kidney is a hallmark of CKD, including lupus nephritis (LN) (1, 6). Our previous study showed that the number of infiltrating immune cells and deposition of immune complexes in the kidney were closely related to the severity of the renal pathological lesions in the LN model mice (1, 7, 8). However, the origin and local regulation of immune cells and autoantibodies in the LN remain unclear.

Effective and prompt immune response to infection or damage is mediated by a well-established immune system. The secondary lymphoid organ (SLO) in adults provides a critical microenvironment for the interaction between immune cells and antigens to propagate an effective adaptive immune response (9). However, during chronic inflammation, ectopic lymphoid tissue can form in inflamed non-lymphoid tissues and demonstrates the most common features of SLO, including stromal chemokine production to attract immune cells, lymphatic vascularization, germinal center (GC) formation, and antibody production. These ectopic lymphoid tissues are called tertiary lymphoid tissues (TLT) and are found in many diseases, including atherosclerosis, persistent infection, cancer, and autoimmune diseases (10–12). TLTs are considered to function as local sites for antigen presentation, perpetuation of antibodies against self-antigens, clonal expansion, and lymphocyte activation (10, 12, 13). The role of TLTs is either beneficial or detrimental depending on the context. Formation of TLTs is beneficial when they clear antigens or pathogens in diseases like viral diseases, cancer, and atherosclerosis (11, 14), while in other diseases such as autoimmune diseases, TLTs are detrimental due to vigorous and sustained response to self-antigen and destruction of normal tissue (11). In the latter context, TLTs may serve as therapeutic targets.

At the beginning of TLT formation in non-lymphoid tissue, resident stromal cells or fibroblasts secrete lymphorganogenic chemokines (LC) (*Cxcl13*) to attract leukocytes for homing (15). Arterial smooth muscle cells (SMCs) also act as lymphoid tissue organizers (LTo) and secrete LC, in atherosclerosis (16). In the skin, the perivascular area is rich in adventitial fibroblasts, and alteration of these fibroblasts leads to the formation of perivascular TLT (17). In addition, aged mice (12 months) subjected to ischemia-reperfusion injury also showed perivascular TLT (15). This study showed that the development of TLT is fully dependent on aging and the presence of acute injury, implying that perivascular fibroblasts only act as LTo. However, there are no reports regarding the role of both vascular and perivascular structures as LTo in TLT development and its correlation with renal histopathology in comparatively younger individuals.

LN is a common complication in young females and the predominant cause of glomerulonephritis (18). Our previous studies used MRL/MpJ-Fas ^lpr/lpr^ (LPR) and BXSB/MpJ-Yaa (Yaa) mice as a systemic lupus erythematosus model to investigate LN and other lupus-related disease pathogenesis (1, 19, 20). Our previous study also showed that female LPR mice developed a well-organized mediastinal fat-associated lymphoid cluster that had more detrimental effects on the lungs than in male LPR mice (20). In the present study, we show a unique perivascular TLT development in the kidney using two LN model mice at different ages and named vasculature-associated lymphoid tissue (VALT). We showed that LPR mice developed inflammation and VALT at an earlier stage (3 months), but VALT became larger and directly correlated with glomerular and tubulointerstitial lesions (GL and TIL) at a later stage (6 months). We also identified the molecular cues responsible for VALT formation, antigen presentation, and GC activity of VALT in the lupus model mice, by using routine histopathological techniques and transcriptomic analysis (TA). Moreover, we identified a therapeutic target to ameliorate LN lesions through ablation of VALT.

## Materials and Methods

### Experimental Mice and Ethical Statement

The authors adhered to ethical guidelines (Institutional Animal Care and Use Committee of the Faculty of Veterinary Medicine, Hokkaido University, approval No. 16-0124) throughout the experiments using experimental mice. LPR mice with their respective control MRL/MpJ (MRL), Yaa, and normal control C57BL/6 (B6) mice were purchased from Japan SLC Inc. (Hamamatsu, Japan). Mice were maintained in special-pathogen-free housing at 1:1 dark and light conditions with *ad libitum* food and water supply.

### Sample Preparation

Mice at 1, 3, and 6 months of age were deeply anesthetized with a mixture of an aesthetic agent as previously described (1), and the kidney slices were fixed with 10% neutral buffer formalin (NBF), 4% paraformaldehyde (PFA), and 2.5% glutaraldehyde (GTA) for histopathological, immunohistochemical, and electron microscopy studies, respectively.

### Dexamethasone (Dex) Administration

Twelve-week-old LPR female mice were divided into two groups: Dex (n = 6) and control group (n = 6). Mice in the Dex group received Dex daily with drinking water and weekly intraperitoneal injections of 0.4 mg/kg body weight, whereas the control group received normal drinking water daily and saline once intraperitoneally every week. Both groups of mice were sacrificed at 22 weeks of age.

### Immunohistochemistry and Immunofluorescence

NBF-fixed paraffin blocks were cut and stained with hematoxylin and eosin (HE), and periodic acid Schiff-hematoxylin (PAS-H) to examine the renal histopathology. Immunodetection of cell markers was performed as previously described (1) for B cells (B220), T cells (CD3), macrophages (Iba1), lymphatic vessels (LYVE-1), high endothelial venule (PNAd), Vimentin, LCs (CCL8, CXCL9 and CXCL13), smooth muscle actin (SMA), follicular dendritic cell (CD21), MHC II, plasma cell (CD138), immunoglobulin (IgG and IGM), and damaged tubules (interleukin 1, family member 6, IL-1F6). The staining conditions are listed in **Supplementary Table 1**.

### Combination of Immunohistochemistry and Aniline Blue Staining

Immunohistochemistry against CXCL13 was performed as previously described. The same tissue sections were stained with aniline blue to colocalize CXCL13 and collagen fibers.

### Histoplanimetry

HE, PAS-H, and immunohistochemistry stained slides from three different planes of the kidney were converted to virtual slides using Nano Zoomer 2.0 RS (Hamamatsu Photonics Co., Ltd.; Hamamatsu, Japan), and the stained area and positive cells were counted using NDP.view2 software (Hamamatsu Photonics Co., Ltd.).

### Reverse Transcription and Real-Time PCR

Total RNA, complementary DNA synthesis, and real-time PCR with Brilliant III SYBR Green QPCR master mix and Mx3000P (Agilent Technologies, La Jolla, CA, USA) was performed as described in our previous study (8). The primers used are listed in **Supplementary Table 2**.

### *In Situ* Hybridization

*In situ* hybridization was performed for *Ccl8*, *cxcl9*, and *cxcl13* as described in our previous study (8). The probes used are listed in **Supplementary Table 2**.

### Microarray Analysis

The excised kidney was treated with RNAlater (Thermo Fisher Scientific) overnight at 4°C. Then, the RNAlater was replaced with TRIzol reagent and stored at –80°C for further analysis (Life Technologies, California, USA). Total RNA was isolated using Trizol reagent following the manufacturer’s instructions. RNA integrity was validated using an Agilent 2100 Bioanalyzer II (Agilent Technologies, Santa Clara, CA, USA), and complementary RNA was synthesized using a Low Input Quick Amp Labeling Kit (Agilent Technologies). Gene expression was analyzed using an Agilent Technologies Microarray Scanner and SurePrint G3 Mouse 8x60K v2.0 (Agilent Technologies), and the raw data normalized by 75Percentile shift (GeneSpring; Agilent Technologies). Toppgene Suite (https://toppgene.cchmc.org/) and Heatmapper (http://www.heatmapper.ca/) were used for gene ontology (GO) analysis and heatmap preparation, respectively, for genes that showed ≥10 folds compared to the control. Microarray data were deposited in a public repository (GSE160488, https://www.ncbi.nlm.nih.gov/geo/query/acc.cgi?acc=GSE160488).

### Scanning Electron Microscopy (SEM)

We modified the sample preparation for SEM for the examination of VALT. Briefly, the mice were perfused with 2.5% GTA through the ventricle. The excised kidney was sliced and fixed with GTA and PFA for 4 and 12 h, respectively, to prepare the paraffin block. The paraffin blocks were cut at 5 mm thickness and mounted on a glass slide. The sections were deparaffinized, fixed, and post-fixed with GTA and osmium tetroxide. The specimen was coated with ion sputter (Hitachi; Tokyo, Japan) for 1 min and examined with an S-4100 SEM (SU 8000, Hitachi) with an accelerated voltage of 10 kV.

### Statistical Analysis

Control and diseased mice were compared using the nonparametric Mann–Whitney *U* test (*P* < 0.05). The Kruskal-Wallis test was used to compare three or more populations, and multiple comparisons were performed using Scheffe’s method when significant differences were observed (*P* < 0.05). The genes that showed expression ≥10 folds compared to the control were selected for statistical analysis, and significant differences between control and disease were calculated by analysis with a 2-tailed Student’s *t* test. Spearman’s rank correlation coefficient (*P* < 0.05) was used to examine the correlation among different parameters.

## Results

### Prevalence of Perivascular Cellular Cluster (PCC) in the Kidney at Different Ages

**Figure 1:**
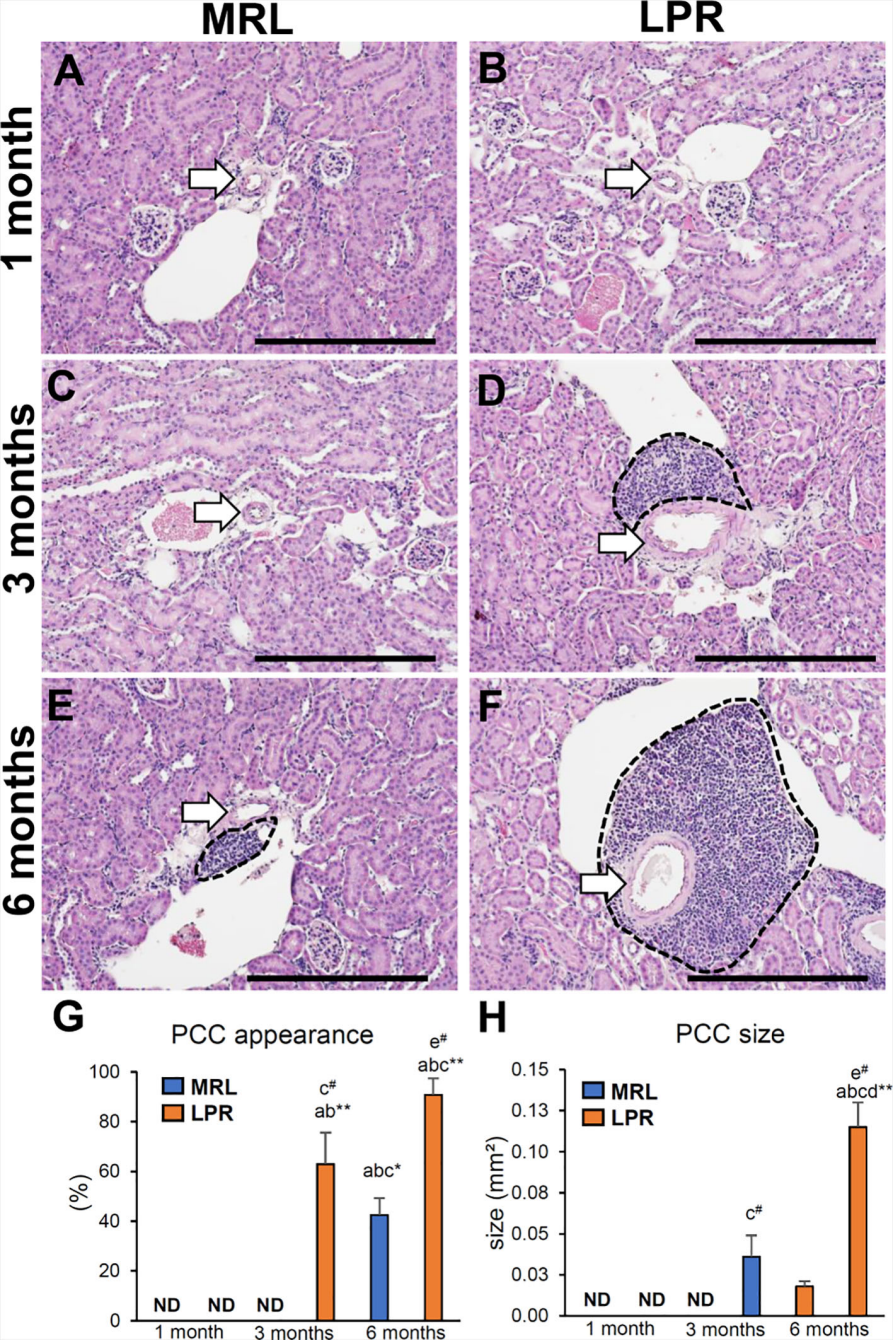
Appearance of PCC at different ages of lupus nephritis model mice kidney. **(A, B)** Absence of PCC near the blood vessel (arrow) in MRL and LPR mice kidney at 1 month of age. **(C)** Absence of PCC near the blood vessel (arrow) in MRL mice kidney at 3 months of age. **(D)** Presence of PCC (dashed area) near the blood vessel (arrow) in LPR mice kidney at 3 months of age. **(E, F)** Presence of PCC (dashed area) near the blood vessel (arrow) in MRL and LPR mice kidney at 6 months of age (HE stain). **(G)** Percentage of PCC appearance in MRL and LPR mice at different ages. **(H)** Size of PCC in MRL and LPR mice at different ages. The values are the mean ± standard error (s.e.). Significant difference from the control in the same age group is indicated by ^#^ (*P* < 0.05, Mann–Whitney *U-*test). Significant differences from the other groups are indicated by * (*P < 0.05, **P < 01, Kruskal-Wallis test followed by Scheffe’s method). n = 4. A, B, C, D, E, and F denote 1 month old MRL, 1 month old LPR, 3 month old MRL, 3 month old LPR, 6 month old MRL, and 6 month old LPR mice, respectively. Bars = 100 *µ*m. PCC, perivascular cellular cluster; HE, hematoxylin and eosin; ND, not detectable.

We examined the prevalence of PCC in lupus prone LPR mice and its corresponding control MRL mice kidney at different ages (**Figure 1**). There was no PCC in any of the mice at 1 month of age (**Figures 1A, B**). At 3 months, PCC was absent in MRL mice but was observed in LPR mice (**Figures 1C, D**). At the later stage (6 months), PCC was observed in both MRL and LPR mouse kidneys, but it was larger in LPR mice (**Figures 1E, F**). We also examined PCC in another lupus-prone mice (Yaa) and normal control B6 mouse kidneys at different ages (**Supplementary Figure 1**). There was no PCC in 1 and 3-month-old B6 and Yaa mice kidneys (**Supplementary Figures 1A–D**). At 6 months of age, PCC was observed in both B6 and Yaa mice kidneys, but it was comparatively larger in Yaa mice (**Supplementary Figures 1E, F**).

The percentage of PCC and its relative size were greater in LPR mice than in MRL mice, at 3 and 6 months of age (**Figures 1G, H**). The percentage of appearance and size of PCC was also higher in LPR mice at 6 months of age compared to the respective controls at the same age and all mice at other ages.

### Cellular Characterization of PCC

**Figure 2:**
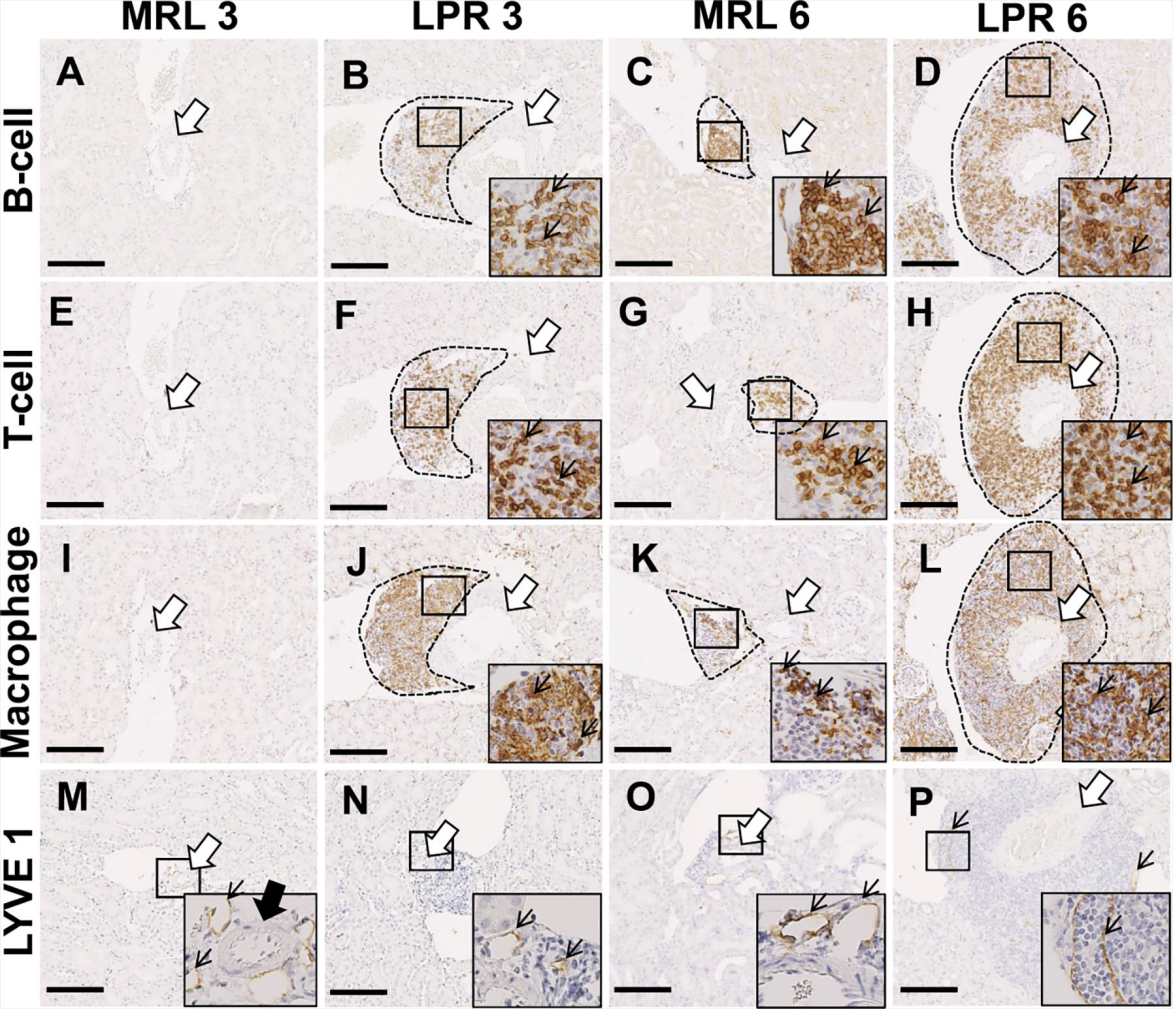
Cellular characterization of PCC at 3 and 6 months of age. **(A–D)** B-cell infiltration (thin arrow) near the blood vessel (arrow) in PCC (dashed area) of MRL and LPR mice kidney at 3 and 6 months of age (IHC). **(E–H)** T-cell infiltration (thin arrow) near the blood vessel (arrow) in PCC (dashed area) of MRL and LPR mice kidney at 3 and 6 months of age (IHC). **(I–L)** Macrophage infiltration (thin arrow) near the blood vessel (arrow) in PCC (dashed area) of MRL and LPR mice kidney at 3 and 6 months of age (IHC). **(M–P)** LYVE1^+^ lymphatic vessels (thin arrow) near the blood vessel (arrow) in PCC (dashed area) of MRL and LPR mice kidney at 3 and 6 months of age (IHC). Bars = 100 *µ*m. PCC, perivascular cellular cluster; IHC, immunohistochemistry.

**Figure 3:**
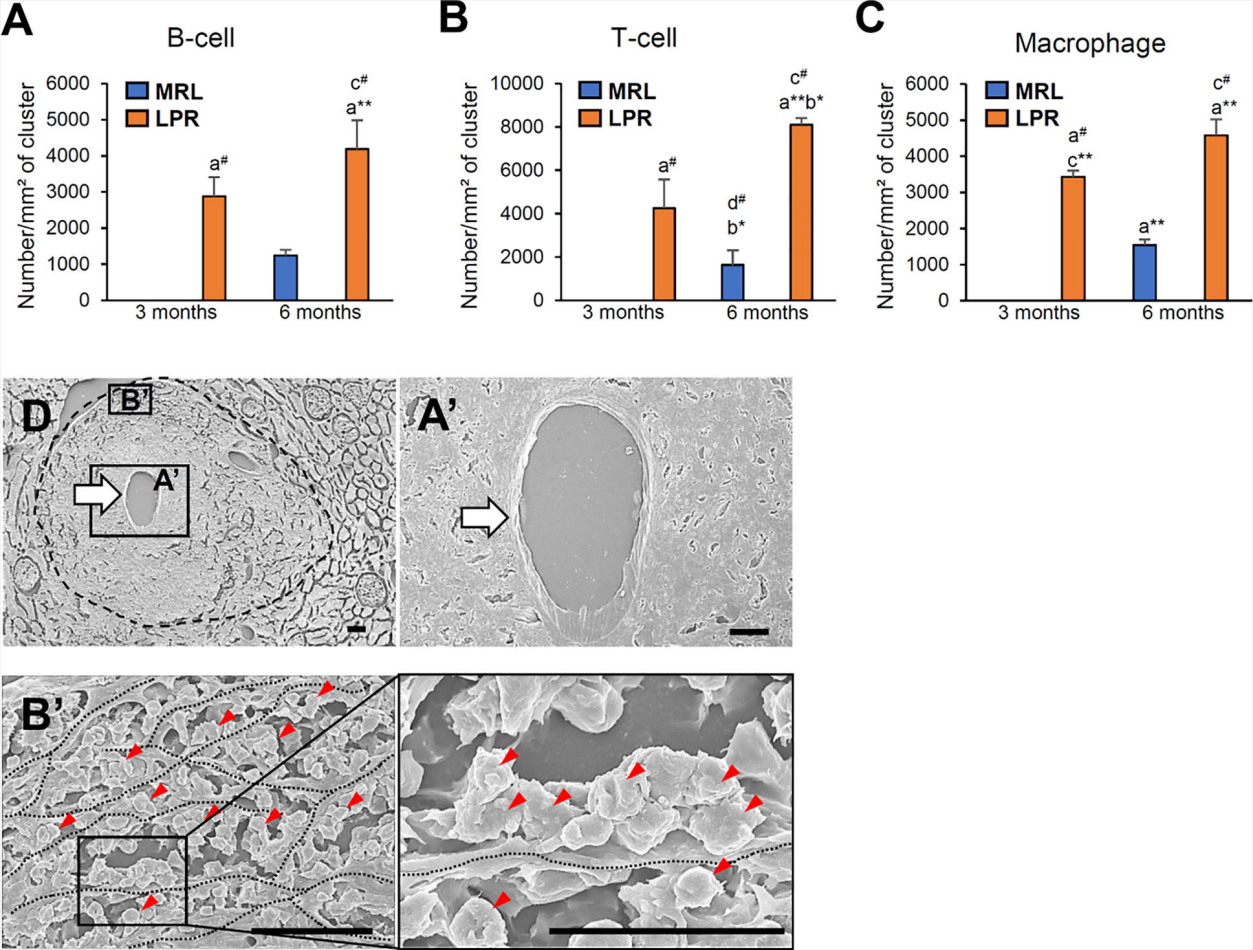
Number of different cellular infiltrates and their cellular organization in PCC. **(A–C)** Number of infiltrating B cells **(A)**, T-cells **(B)**, and macrophages **(C)** in PCC of MRL and LPR mice kidney at 3 and 6 months of age. The values are the mean ± s.e. Significant difference from the control in the same age group is indicated by ^#^ (*P* < 0.05, Mann–Whitney *U-*test). Significant differences from the other groups are indicated by * (*P < 0.05, **P < 01, Kruskal-Wallis test followed by Scheffe’s method). n = 4. A, B, C, and D denote 3 months-old MRL, 3 months-old LPR, 6 months-old MRL, and 6 months-old LPR mice, respectively. **(D)** A PCC (encircled dashed area) surrounds the artery (thick arrow), which has no abnormality in wall (A’, thick arrow). Immune cells (red arrowheads) are arranged in chambers made by stromal anastomoses (B’) (dotted line). Immune cells (red arrowheads) are attached to the stromal wall (inset) (dotted line). SEM. Bars = 10 µm. PCC, perivascular cellular cluster; SEM, scanning electron microscopy.

We characterized the PCC cluster cells by immunohistochemistry using different cell markers (**Figure 2**). We observed that PCCs were composed of B-, T-cells, and macrophages (**Figures 2A–L**). Next, we examined the presence of high endothelial venules (HEV) and lymphatic vessels in or around the clusters. We did not observe any high HEV either in or around the PCC in either MRL or LPR mice (data not shown). However, LYVE-1^+^ lymphatic vessels accompanied by arteries were found in MRL mice at 3 months of age, though no cellular cluster was observed (**Figure 2M**), whereas lymphatic vessels were observed in and around PCC in 6 month old MRL, and 3 and 6 month old LPR mice (**Figures 2N–P**). The number of B-, T-cells, and macrophages in the cluster was higher in LPR mice than in control mice at all ages, but highest in LPR mice at 6 months of age (**Figures 3A–C**). We also examined PCCs by SEM (3D). No abnormality of the vascular wall was observed, and cluster cells were arranged in chambers made by stromal anastomoses (**Figures 3D-A’, B’**).

### Evaluation of LC and Molecules Responsible for Lymphoid Tissue Formation

**Figure 4:**
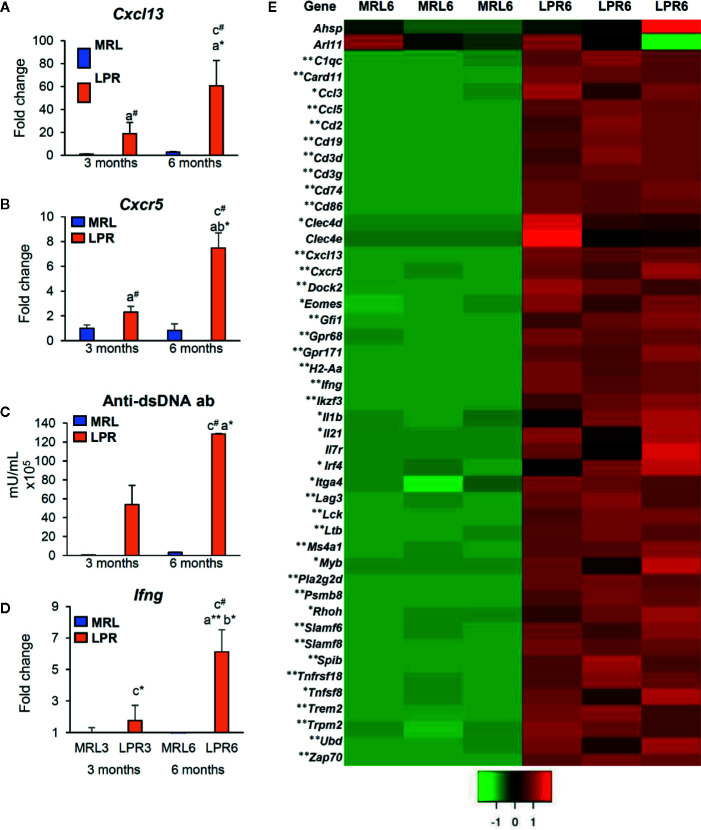
Expression of lymphorganogenic chemokines, cytokines, and costimulatory molecules. **(A, B)** Relative *mRNA* expression of *Cxcl13* **(A)** and *Cxcr5* **(B)** in MRL and LPR mice at 3 and 6 months of age (qPCR). **(C)** Serum anti-dsDNA antibody in MRL and LPR mice at 3 and 6 months of age (ELISA). **(D)** Relative mRNA expression of *Ifng* in MRL and LPR mice at 3 and 6 months of age (qPCR). The expression levels were normalized to the levels of *actb*. The values are the mean ± s.e. Significant difference from the control in the same age group is indicated by ^#^ (*P* < 0.05, Mann–Whitney *U-*test). Significant differences from the other groups are indicated by * (*P < 0.05, **P < 01, Kruskal-Wallis test followed by Scheffe’s method). n = 4. A, B, C, and D denote 3 months old MRL, 3 months old LPR, 6 months old MRL, and 6 months old LPR mice, respectively. **(E)** Expression of transcripts for lymphorganogenic chemokines, cytokines, co-stimulatory molecules, and their receptors in MRL and LPR mice kidney at 6 months of age. Microarray analysis, significant difference from the control is indicated by * (*P < 0.05, **P < 01, 2-tailed Student’s *t* test). n = 3. qPCR, Quantitative PCR; dsDNA ab, double strand DNA antibody; ELISA, enzyme-linked immunosorbent assay; *Ifng*, interferon gamma.

**Figure 5:**
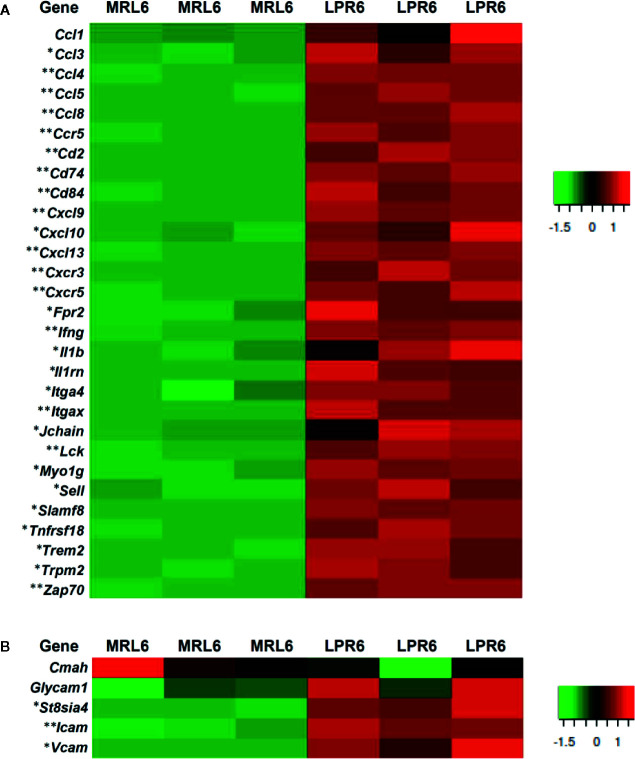
Expression of different transcripts related to leukocyte migration, high endothelial venules forming molecules, and adhesion molecules. **(A)** Gene transcripts related to leukocyte migration in MRL and LPR mice at 6 months of age. Microarray analysis, significant difference from the control is indicated by * (*P < 0.05, **P < 01, 2-tailed Student’s *t* test). n=3. **(B)** Gene transcripts related to high endothelial venules forming molecules and adhesion molecules in MRL and LPR mice at 6 months of age. Microarray analysis, significant difference from the control is indicated by * (*P < 0.05, **P < 01, 2-tailed Student’s *t* test). n=3.

As the PCC consists of B-, T-cells, and macrophages and has lymphatic vessels, we examined the principle LC (*Cxcl13*) and its receptor (*Cxcr5*) responsible for lymphoid tissue formation in 3 and 6-month-old mice to distinguish PCC from the perivascular cuffing (**Figure 4**). The expression of both *Cxcl13* and *Cxcr5* was upregulated in LPR mice compared to their respective controls (**Figures 4A, B**). Higher levels of serum anti-dsDNA antibody were found in the LPR mice compared to the control (**Figure 4C**). Examination of the inflammatory cytokines in the whole kidney revealed upregulation of *Ifng* compared to their respective controls, only in LPR mice (**Figure 4D**). Importantly, serum anti-dsDNA antibody was correlated with *Cxcl13* expression, PCC appearance, and *Ifng* expression level (**Supplementary Figures 2A–C**). Moreover, *Ifng* expression was correlated with the expression level of *Cxcl13* and the appearance of PCC (**Supplementary Figures 2D, E**). Since we observed a higher prevalence of large PCC, upregulation of LCs, serum anti-dsDNA antibody, and inflammatory cytokines in LPR mice at 6 months of age, we performed TA on kidneys from MRL and LPR mice at this age and selected genes that showed >10-fold upregulation, for further analysis, to correlate with our histologic findings. GO analysis using upregulated transcripts revealed an LPR kidney expression signature associated with lymphoid tissue formation (GO:0048534, *P* = 4.14e^-25^) (**Figure 4E**). GO also identified an expression signature associated with leukocyte migration (GO:0050900, *P* = 9.24e^-18^) (**Figure 5A**). Moreover, TA analysis revealed upregulation of HEV forming molecules (*Glycam* and *St8sia4*) and adhesive molecules (*Icam* and *Vcam*) (**Figure 5B**).

### Localization of Structures Producing LCs for Lymphoid Tissue Formation in PCC

**Figure 6:**
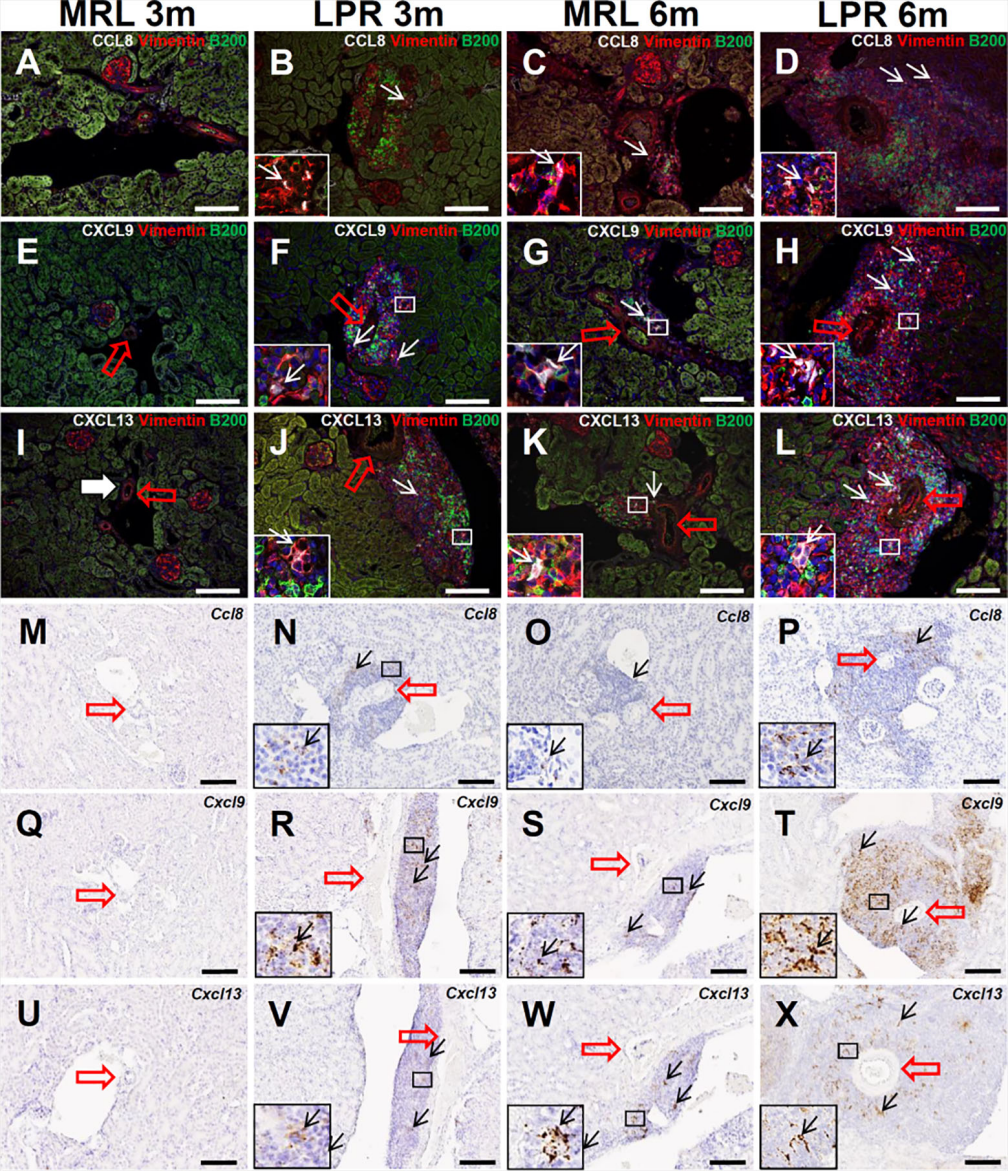
Localization and expression of lymphorganogenic chemokines in PCC. **(A–D)** Colocalization (arrows) of CCL8 with vimentin and B200^+^ B cells in PCC near the blood vessel (thick arrow) of MRL and LPR mice at 3 and 6 months of age (IF stain). **(E–H)** Colocalization (arrows) of CXCL9 with vimentin and B200^+^ B cells in PCC near the blood vessel (thick arrow) of MRL and LPR mice at 3 and 6 months of age (IF stain). **(I–L)** Colocalization (arrows) of CXCL13 with vimentin and B200^+^ B cells in PCC near the blood vessel (thick arrow) of MRL and LPR mice at 3 and 6 months of age (IF stain). **(M–P)** Expression (arrows) of *Ccl8* in PCC near the blood vessel (thick arrow) of MRL and LPR mice at 3 and 6 months of age (ISH). **(Q–T)** Expression (arrows) of *Cxcl9* in PCC near the blood vessel (thick arrow) of MRL and LPR mice at 3 and 6 months of age (ISH). **(U–X)**. Expression of *Cxcl13* in PCC near the blood vessel (thick arrow) of MRL and LPR mice at 3 and 6 months of age (ISH). Bars = 100 µm. PCC, perivascular cellular cluster; IF, immunofluorescence; ISH, *in situ* hybridization.

We aimed to characterize the 3 most abundantly expressed chemokines (*Ccl8*, *Cxcl9*, and *Cxcl13*) in TA analysis related to leukocyte chemotaxis and migration (**Supplementary Data Sheet 1**). Therefore, we examined the protein localization of chemokine CCL8, CXCL9, and -13 in PCC. CCL8 localization was not observed in MRL mice kidney at 3 months of age, but its localization was observed with vimentin in the PCC of LPR mice at 3 months of age, and in MRL and LPR mice at 6 months of age (**Figures 6A–D**). CXCL9 localization was not observed in MRL mice kidney at 3 months of age, but its localization was observed with vimentin in the PCC of LPR mice at 3 months of age, and in MRL and LPR mice at 6 months of age (**Figures 6E–H**). Similarly, CXCL13 localization was not observed in MRL mice kidney at 3 months of age, but its localization was observed with vimentin in the PCC of LPR mice at 3 months of age, and in MRL and LPR mice at 6 months of age (**Figures 6I–L**). *Ccl8*, *Cxcl9*, and *Cxcl13* expression was not observed in MRL mice kidney at 3 months of age, but its expression was observed in PCC of LPR mice at 3 months of age, and in MRL and LPR mice at 6 months of age (**Figures 6M–X**).

### Vascular and Perivascular Structures Produced LCs

**Figure 7:**
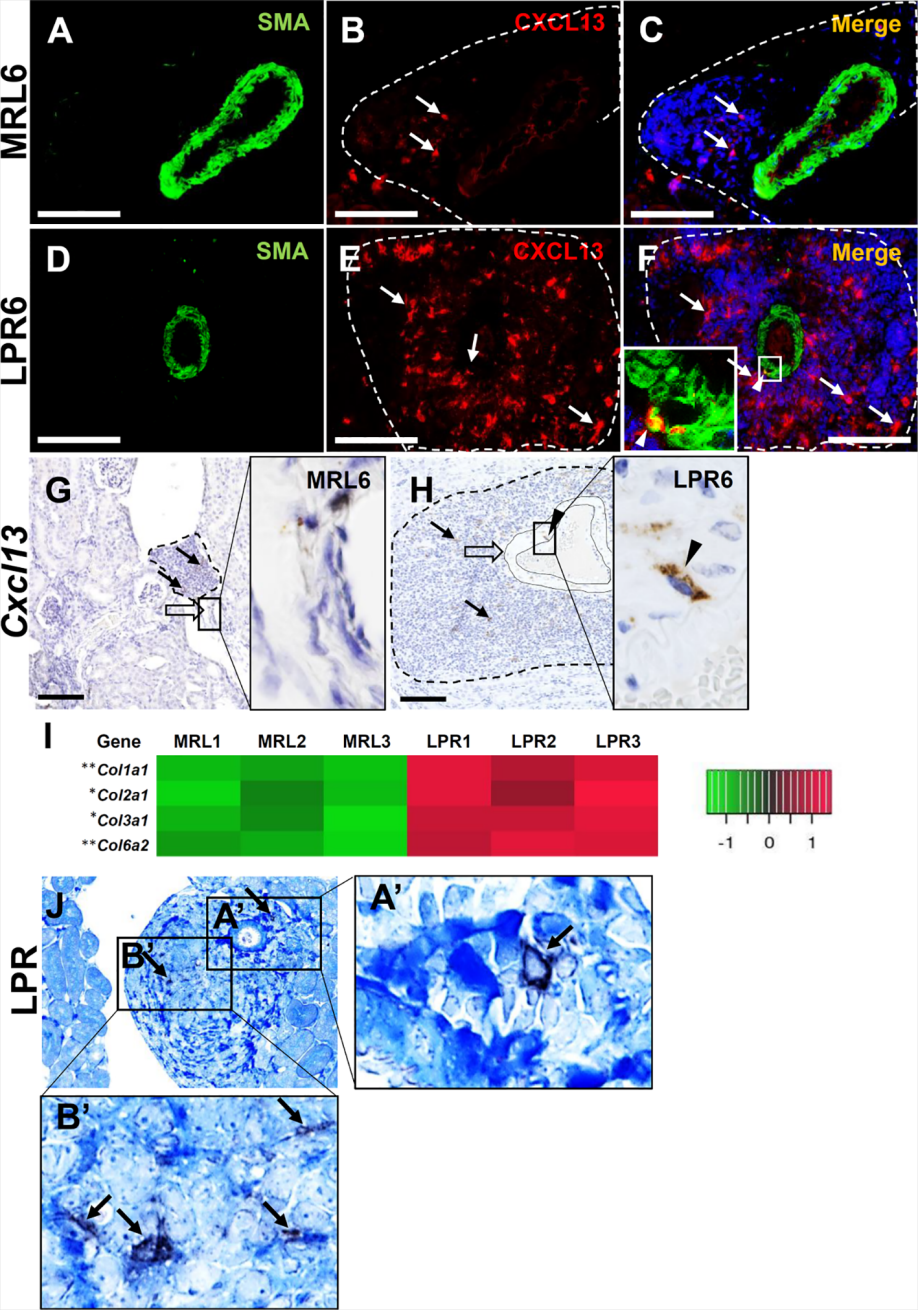
Lymphorganogenic chemokine localization and expression in vascular and perivascular structure. **(A–C)** CXCL13 (arrow) did not colocalize with SMA^+^ smooth muscle cells of the arteries in MRL mice at 6 months of age (IF stain). **(D–F)** CXCL13 (arrow) colocalized (arrowhead) with SMA^+^ smooth muscle cell of the artery in LPR mice at 6 months of age (IF stain). **(G, H)** *Cxcl13* was not expressed at the arterial wall of MRL mice **(G)** at 6 months of age but was expressed at the arterial wall of LPR mice at the same age **(H)** (ISH). **(I)** Transcripts for different genes of collagen in MRL and LPR mice at 6 months of age. Microarray analysis, significant difference from the control is indicated by * (*P < 0.05, **P < 01, 2-tailed Student’s *t* test). n = 3. **(J)** Colocalization of CXCL13 (arrow) with collagen fiber in PCC of LPR mice at 6 months of age (IHC and aniline blue stain). Bars = 100 µm. PCC, perivascular cellular cluster; IF, immunofluorescence; ISH, *in situ* hybridization; IHC, immunohistochemistry.

We also examined the role of vascular and perivascular structures in the production of LCs for lymphoid tissue formation. Protein localization and expression for *Ccl8* and *Cxcl9* in vascular structures was not observed in mice at any stage (data not shown). CXCL13 protein was not observed in MRL mice vascular structures at 6 months of age, but it was co-localized with SMC in the tunica media of the arteries in LPR mice at the same age (**Figures 7A–F**). This result was also confirmed by *in situ* hybridization, which revealed that SMC of the vascular tunica media expressed *Cxcl13* in LPR mice at 6 months of age (**Figures 7G, H**). TA showed upregulation of different collagen fibers in the kidney of LPR mice, compared to that in MRL mice (**Figure 7I**). The combination of immunohistochemistry for CXCL13 and aniline blue staining revealed colocalization of collagen fiber and CXCL13 (**Figures 7J-A’, B’**) in LPR mice at 6 months of age. PCC was termed as vasculature-associated lymphoid tissue (VALT) hereafter, since vascular and perivascular structures produce LCs for PPC formation.

### Antigen Presentation in VALT

**Figure 8:**
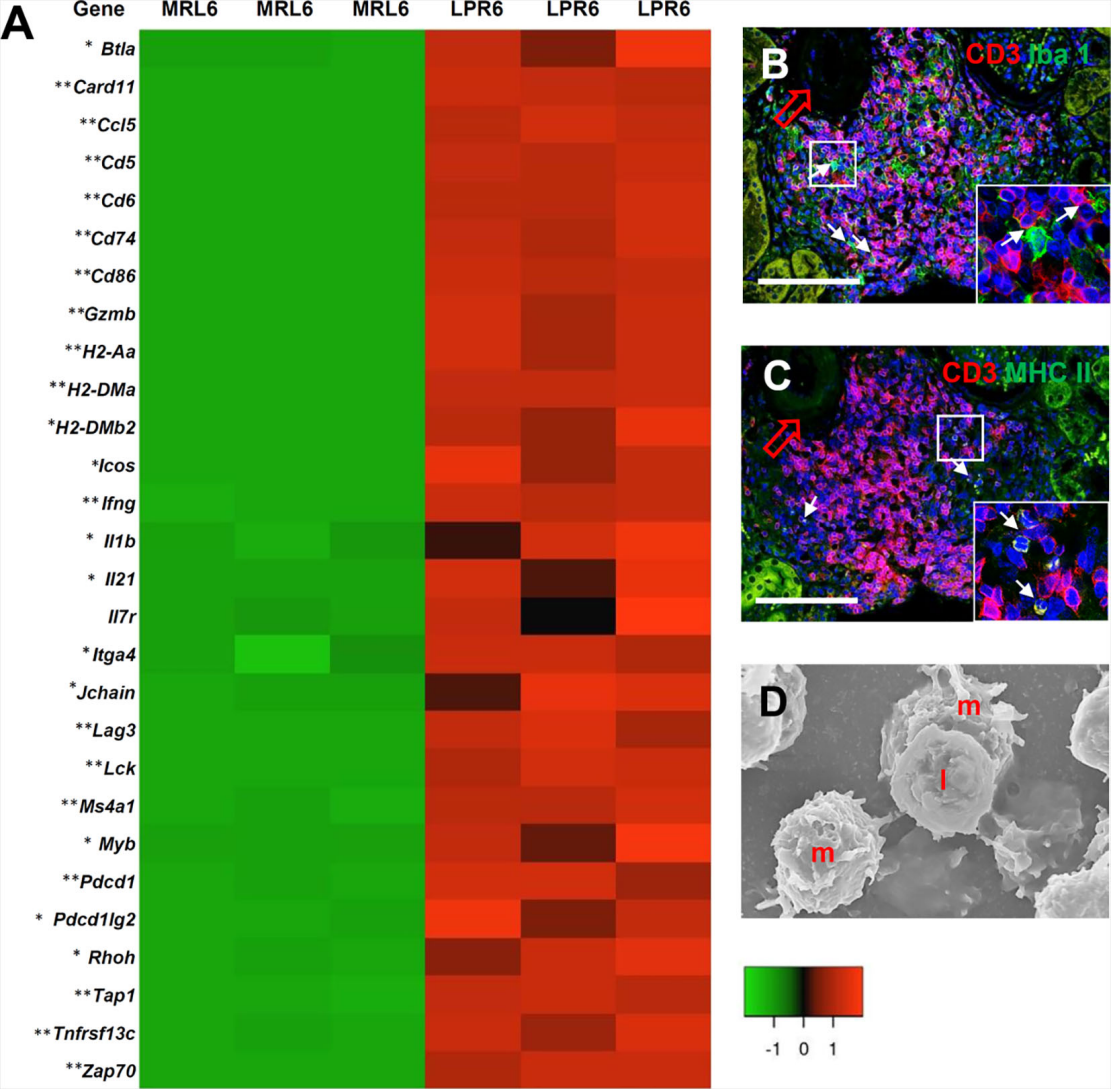
Antigen presentation and lymphocyte activity in VALT. **(A)** Expression levels of different chemokines, receptors, and costimulatory molecules for antigen presentation and cellular activity in VALT of MRL and LPR mice at 6 months of age. Microarray analysis, significant difference from the control is indicated by * (*P < 0.05, **P < 01, 2-tailed Student’s *t* test). n = 3. **(B)** CD3^+^ lymphocytes are localized adjacent to Iba1^+^ macrophages (arrows) in VALT of LPR mice at 6 months of age (IF stain). **(C)** CD3^+^ lymphocytes are localized near MHC II molecules on antigen-presenting cells (arrows) in VALT of LPR mice at 6 months of age (IF stain). **(D)** Lymphocytes **(I)** are juxtaposed on macrophages (m) in VALT of LPPR mice at 6 months of age (SEM). Bars = 100 µm **(B, C)** and 5 µm **(D)**. VALT, vasculature-associated lymphoid tissue; IF, immunofluorescence; SEM, scanning electron microscopy.

GO analysis revealed several transcripts for genes related to immunological synapses (GO:0001772, *P* = 9.03e^-09^), major histocompatibility complex (MHC) class II protein binding (GO:0042289, *P* = 2.38e^-04^), MHC class II protein complex binding (GO:0023026, *P* = 4.56e^-06^), antigen binding (GO:0003823, *P* = 258e^-04^), and positive regulation of T cell activation (GO:0050870, *P* = 1.32e^-17^) (**Figure 8A**). We also evaluated VALT infiltrates for evidence of antigen presentation and found colocalization of CD3^+^ lymphocytes and Iba1^+^ macrophages as well as a combination of CD3^+^ lymphocytes and MHC II molecules on antigen-presenting cells, consistent with antigen presentation to T lymphocytes in VALT (**Figures 8B, C**). Moreover, SEM revealed juxtaposition of lymphocytes with antigen-presenting cells (**Figure 8D**).

### VALT in Lupus-Prone Mice Mimics the Lymphoid Follicle With GC Activity

**Figure 9:**
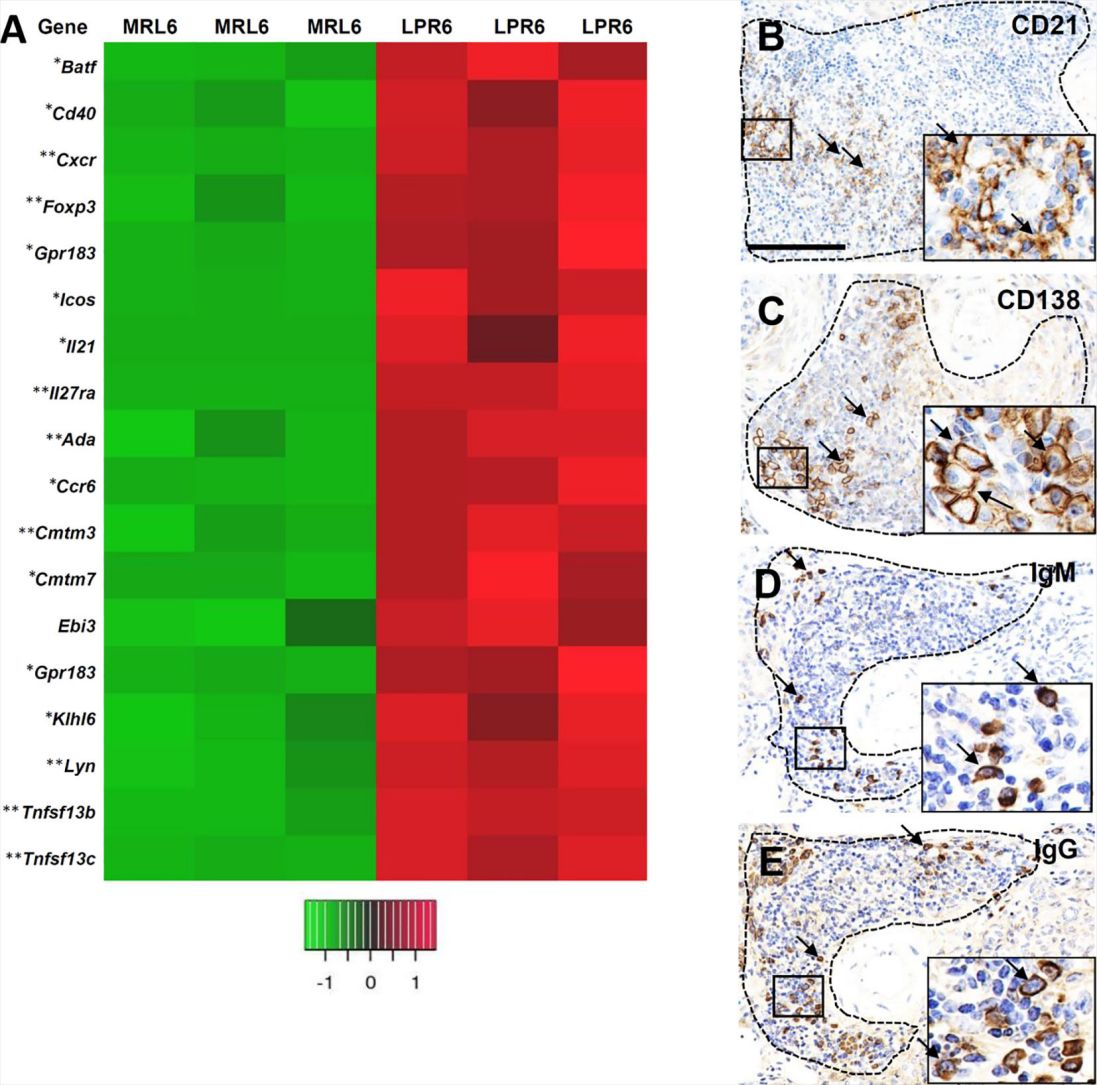
VALT shows lymphoid follicular and germinal center activity. **(A)** Different gene transcripts related to lymphoid follicular and germinal center activity in MRL and LPR mice at 6 months of age. Microarray analysis, significant difference from the control is indicated by * (*P < 0.05, **P < 01, 2-tailed Student’s *t* test). n = 3. **(B)** CD21^+^ follicular dendritic cells (arrows) in VALT of LPR mice at 6 months of age (IHC). **(C)** CD138^+^ plasma cells (arrows) in VALT of LPR mice at 6 months of age (IHC). **(D)** IgM^+^ cells (arrows) in VALT of LPR mice at 6 months of age (IHC). **(E)** IgG^+^ cells (arrows) in VALT of LPR mice at 6 months of age (IHC). Bars = 100 µm. VALT, vasculature-associated lymphoid tissue; IHC, immunohistochemistry.

Following determination of the nature and organization of cellular infiltrates in VALT, we characterized the individual cells and transcription factors to determine whether VALT in lupus-prone mice mimics lymphoid follicle with GC activity (**Figure 9**). Higher expression of transcripts for T-cell plasticity and acquisition of T-follicular helper cells (T_FH_), including *Batf*, *Cd40, Cxcr5*, *Foxp3*, *Gpr183*, *Icos, Il21*, and *Il27ra* were observed in LPR kidney (**Figure 9A**). Higher expression of transcription factors for B-cell receptor signaling, B-cell survival, maturation, proliferation, GC activity, and antibody class switching (*Ada*, *Cmtm3*, *Cmtm7*, *Ccr6*, *Ebi3*, *Gpr183*, *Klhl6*, *Lyn*, *Tnfrsf13b*, and *Tnfrsf13c*) was observed in the kidney of LPR mice (**Figure 9A**). CD21^+^ follicular dendritic cells (FDC), CD138^+^ plasma cells, IgM, and IgG^+^ cells were observed in VALT of LPR mice kidney at 6 months of age (**Figures 9B–E**). Moreover, upregulation of transcripts for different autoantigens and BrdU^+^ proliferating cells was also observed (**Supplementary Figures 3A, B**).

### VALT in Lupus-Prone Mice Correlated With Renal Lesions

**Figure 10:**
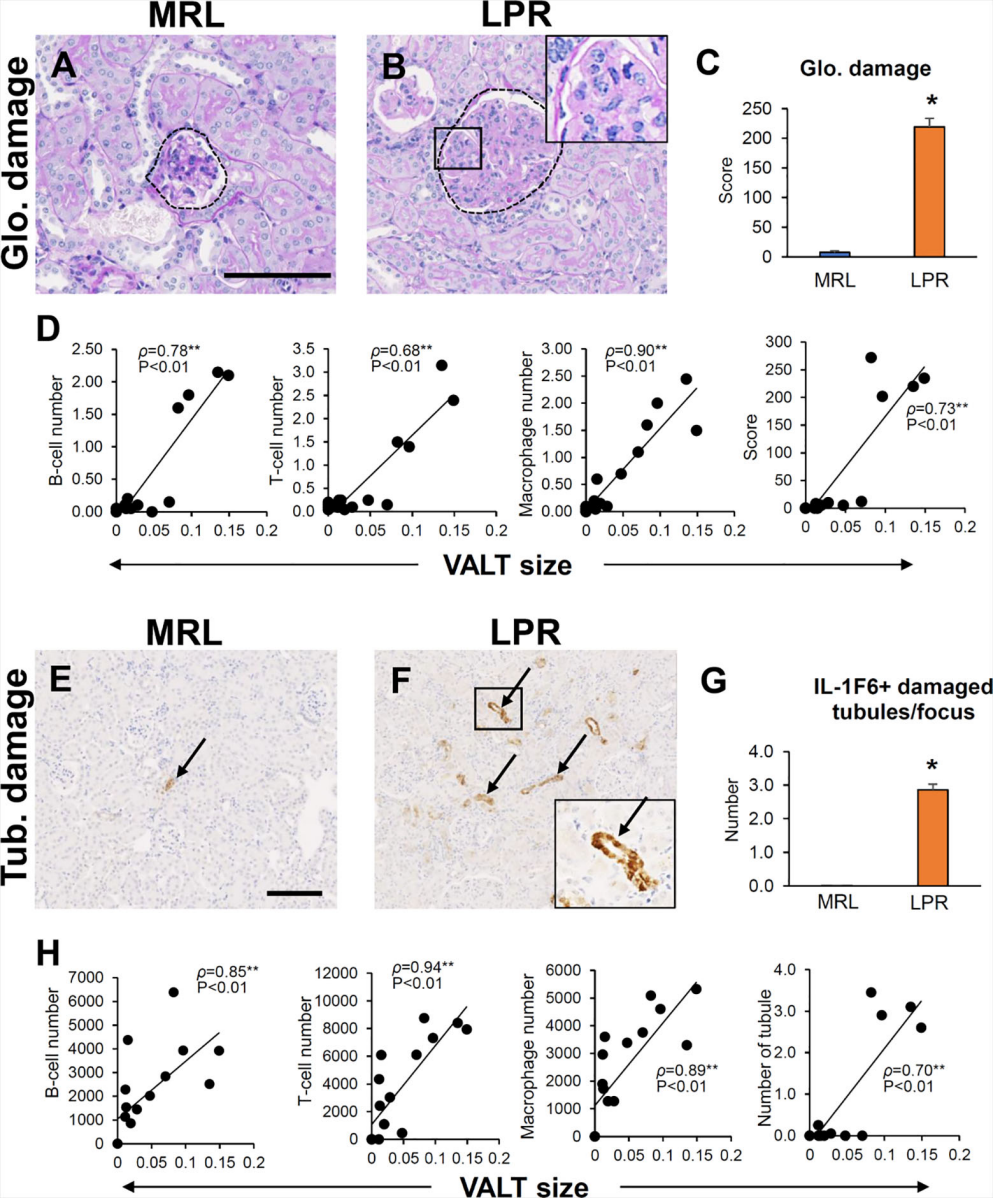
Association of VALT and renal pathology. **(A, B)** Glomerular damage in MRL and LPR mice at 6 months of age (PAS-H stain). **(C)** Glomerular damage score in MRL and LPR mice at 6 months of age. **(D)** Correlation between VALT size and GL parameters. **(E, F)** IL-1F6^+^ damaged tubules in MRL and LPR mice at 6 months of age. **(G)** Number of IL-1F6^+^ damaged tubules in MRL and LPR mice at 6 months of age. **(H)** Correlation between VALT size and TIL parameters. The values are the mean ± s.e. *significantly different from control mice (Mann-Whitney *U*-test, *P* < 0.05); n = 4. **P < 0.01, Spearman’s rank correlation coefficient, n = 16. Bars = 100 µm. VALT, vasculature-associated lymphoid tissue; PAS-H, periodic acid *Schiff*-hematoxylin; IHC, immunohistochemistry; GL, glomerular lesion; TIL, tubulointerstitial lesion; glo, glomerular; Tub, tubular.

Infiltrating B-, T-cells, and macrophages in the glomerulus were higher in 6-month-old LPR mice (Data not shown), who also developed glomerulosclerosis. Semiquantitative glomerular damage score was higher in LPR mice than in MRL mice at the same age (**Figures 10A–C**). The size of VALT was correlated with infiltrating B-, T-cells, and macrophages in the glomerulus and glomerular damage score (**Figure 10D**). Infiltrating B-, T-cells, and macrophages in the tubulointerstitium was higher in LPR mice at 6 months of age (Data not shown). IL-1F6^+^ damaged tubules were more abundant in LPR mice at 6 months of age than in MRL mice at the same age (**Figures 10E–G**). The size of VALT was also correlated with infiltrating B-, T-cells, and macrophages in tubulointerstitium and IL-1F6^+^ damaged tubules (**Figure 10H**).

### Ablation of VALT Using Immunosuppressive Drugs

**Figure 11:**
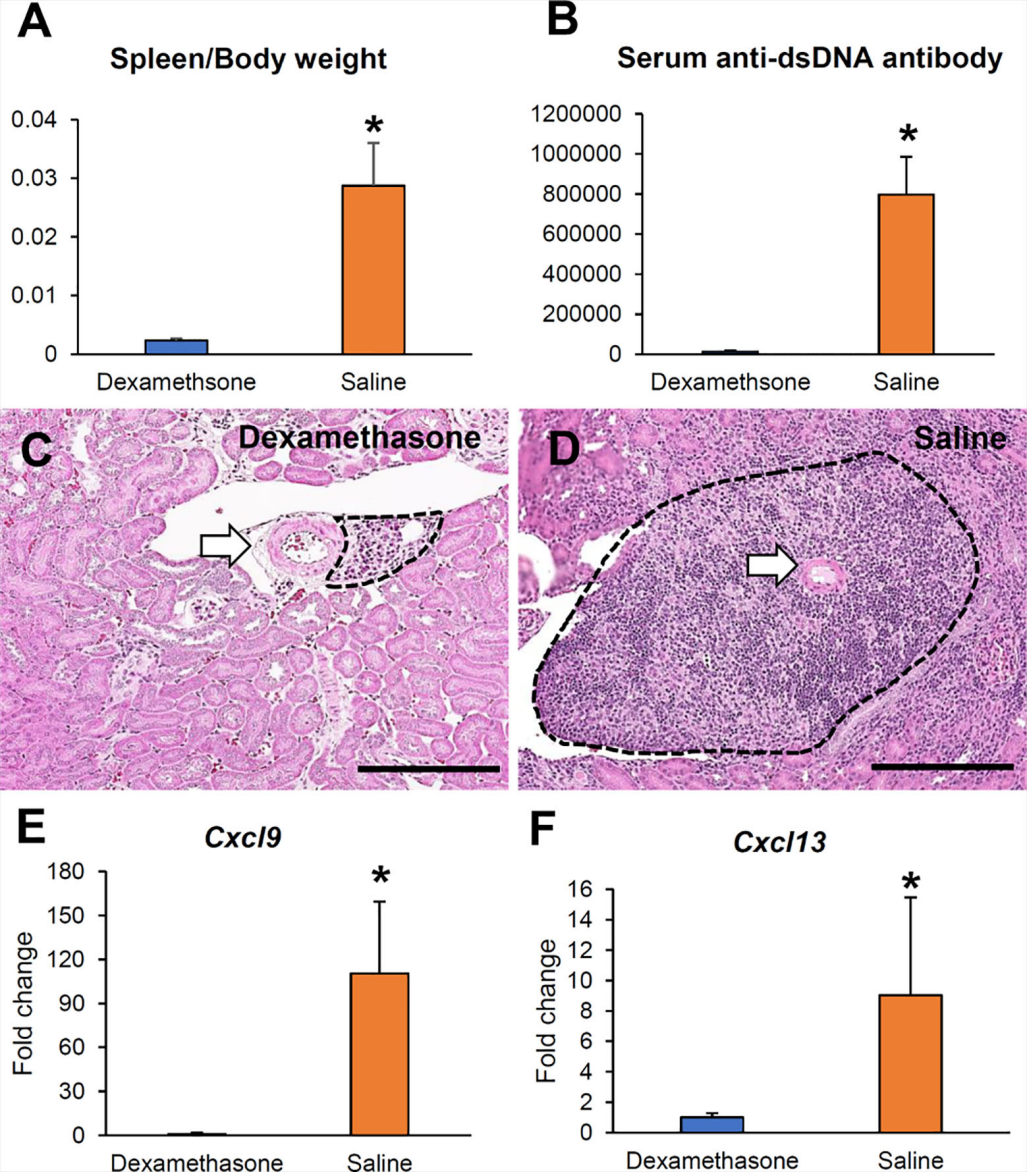
Reduction of VALT size by dexamethasone. **(A, B)** Serum anti-dsDNA antibody level and spleen to body weight ratio in the Dex and saline groups. **(C, D)** Smaller VALT in the Dex **(C)** group compared to the control group **(D)**. HE stain. Bars = 100 µm. **(E, F)** *Cxcl9* and *-13* expression levels in the Dex and control groups. qPCR. The expression levels were normalized to the levels of *Actb*. The values are the mean ± s.e. *, significantly different from control mice (Mann-Whitney *U*-test, *P* < 0.05); n = 4. Dex, dexamethasone; HE, hematoxylin and eosin; qPCR, quantitative PCR.

We administered Dex as an immunosuppressive drug to ablate VALT. The Dex group showed lower serum anti-dsDNA antibody and spleen to body weight ratio compared to the saline control (**Figures 11A, B**). The Dex group had almost no or reduced size VALT compared to the control group (**Figures 11C, D**). Moreover, *Cxcl9* and *Cxcl13* expression was significantly reduced in the Dex group compared to that in the control (**Figures 11E, F**).

## Discussion

Our present study clarified the development of VALT in LN using two widely used lupus-prone model mice (LPR and Yaa) at different ages. This study shows that VALT develops at an earlier stage (3 months) in lupus-prone mice only. VALT development in LN is associated with chemokines, cytokines, and costimulatory molecules related to typical lymphoid tissue development and organization. Interestingly, lupus-prone mice showed large VALT, which was associated with renal histopathology at 6 months of age, while the respective control mice developed small VALT in the absence of renal pathology.

We first determined the age at which VALT first appears and measured its average size at different ages. At the earlier stage, VALT was found only in lupus-prone mice (**Figure 1**). While lupus model mice developed large VALT at 6 months of age, the control MRL mice developed small VALT (**Figure 1**). At these stages, cellular characterization of VALT allowed distinction from perivascular cuffing. VALT was found to compose of B-, T-cells, and macrophages, and was accompanied by lymphatic vessels (**Figure 2**). Moreover, SEM revealed that this did not result from arterial damage but rather from the presence of different types of cells in the stromal chambers, as observed in typical lymphoid organs (**Figure 3**). These results suggest that VALT appearance and development might be associated with inflammatory conditions, aging, and developing the features of TLT. Importantly, previous studies showed that TLT formation was triggered by chronic inflammation and autoimmunity (9, 21). Therefore, we examined the expression level of major LC (*Cxcl13*) (22) and its receptor (*Cxcr5*), serum anti-dsDNA antibody levels, and inflammatory cytokines in kidneys from MRL and LPR mice at both stages and found that their expression was higher in LPR mice kidney compared to MRL at both stages (**Figure 4**). Moreover, serum anti-dsDNA antibody and *Ifng* expression correlated with *Cxcl13* expression and percentage of VALT appearance (**Supplementary Figure 2**). These results proved that these VALTs are TLT and result from autoimmune condition.

We further examined how VALT was formed around the blood vessels. RNA sequencing has proven to be a powerful technique for identifying the multifaceted and complex transcriptional activity that is responsible for TLT formation (23). Therefore, we performed TA using kidney tissues from LPR and MRL mice at this age to reveal the chemokines, cytokines, and costimulatory molecules responsible for VALT formation. GO analysis revealed highly upregulated genes, consistent with TLT formation (**Figure 4**). Typical lymphoid tissue is usually developed by the interaction of hematopoietic lymphoid tissue inducer (LTi) and mesenchymal origin LTo (22). LTi cells expressing CXCR5 and IL7R chemokines accumulate in newly formed lymphoid tissues in response to local production of CXCL13 and IL7 by LTo cells (24). Therefore, our TA clearly revealed that hematopoietic LTi cells are responsible for VALT formation.

We examined the localization and *in situ* expression of highly expressed chemokines in VALT to identify their local sources. We observed that CCL8, CXCL9, and CXCL13 proteins were colocalized with vimentin (**Figure 6**). Moreover, *in situ* expression of *Ccl8*, *Cxcl9*, and *Cxcl13* was observed in the interstitial space among infiltrating cells in VALT. These results indicate that mesenchymal-originated cells such as stromal cells are responsible for the expression of LCs and act as LTo for VALT. Moreover, our TA also showed higher expression of adhesion molecules (*Icam* and *Vcam*), which facilitates the retention of cells in VALT (**Figure 5**).

Cellular clusters were confined only to the perivascular area. It is not surprising that the space between the artery and the vein harbored clusters of infiltrating cells, as it provides space in this solid organ. However, the formation of these clusters was determined by examining the role of vascular and perivascular structures as LTo (**Figure 7**). We observed the colocalization of CXCL13 with SMC of arterial tunica media, by immunohistochemistry and *in situ* hybridization. In addition, TA also showed higher transcription for many collagen-related genes, including *col1*, *-2*, -*3*, and -*6*. Therefore, we examined the role of these perivascular collagen fibers as LTo. Surprisingly, we found colocalization of CXCL13 with perivascular collagen fibers. Taken together, SMC and perivascular collagen fibers also produced LCs to attract leukocytes. Therefore, vascular SMCs and collagen fibers act as LTo in addition to perivascular stromal cells in the formation of VALT. This VALT is different from the recently characterized lymph node-like structures on the renal pelvic wall in lupus nephritis model mice kidney because VALT developed separately at an early stage around the large blood vessels and had different lymphatic vasculatures and lymphoid tissue organizers (25). Moreover, serial sectioning examination confirmed that VALT is connected to the lymphoid cluster on the renal pelvic wall only at the late stage.

Usually, TLTs are vascularized with high endothelial venules and lymphatic vessels for lymphocyte trafficking (26). However, we observed only LYVE1^+^ lymphatic vessels in the VALT. Although PNAd expression in VALT could not be detected, a higher expression for transcripts of *Glycam 1* and *St8sia4* (**Figure 5**) were detected, indicating that PNAd constituting proteins undergo local posttranslational modification, suggesting that leukocyte trafficking into VALT may also be directed by elements, in addition to vasculatures. Moreover, our previous study also showed peritubular capillary injury and extravasation of inflammatory cells in the tubulointerstitial space in LN (27). This indicates that extravasated inflammatory cells also have the potential to be attracted by LCs and contribute to the VALT population in LN.

TLTs function as local sites for the generation of antibodies as a result of local antigen presentation, lymphocyte activation, and maturation (28). In this study, we evaluated the antigen presentation, GC activity, and antibody production in VALT to clarify its functional activity. We evaluated the infiltrates in VALT to examine antigen presentation and found colocalization of CD3 and Iba1^+^ cells as well as CD3 and MHC-II, indicating local antigen presentation to T cells. Similarly, SEM observation also revealed colocalization of lymphocytes and macrophages (**Figure 8**). Moreover, GO demonstrated the enrichment of genes related to antigen presentation, lymphocyte activation, and immunological synapse (**Figure 8**). *Il2rb2* stimulates T cell proliferation towards Th1 by enhancing *Ifng* through *Il27*. T_FH_ cells are characterized by their surface markers *Cxcr5* and *Icos* and by the expression of *Il21* upon stimulation (29, 30). TA revealed higher transcription for *Cxcr5*, *Icos*, *Il12rb2*, *Il27*, *Il21*, and *Cd40* (**Figure 9**). Therefore, we concluded that helper T cells become T_FH_ cells in VALT and promote formation of GC, where B cells rapidly differentiate into antibody-producing plasma cells with greater affinity. TA also revealed several genes which support the GC activity, including *klhl6* (B cell receptor transduction signals), *Ada* (GC B-cell survival), *Tnfsf13b* (BAFF), *and Tnfrsf13c* (BAFF receptor). Moreover, FDC plays a crucial role in B-cell activation and affinity maturation through the continuous presentation of antigens to B cells (31). We detected CD21^+^ FDC in VALT (**Figure 9**) and identified several autoantigens in the kidney of LPR mice (**Supplementary Figure 3**). Therefore, we concluded that FDCs continuously present autoantigens to B-cells for the perpetuation of local antibodies. Importantly, we observed CD138^+^ plasma cells as well as IgM and IgG antibodies at the periphery of VALT. This result is consistent with previous results that showed that TLT was associated with autoantibody production in autoimmune disease (21, 32, 33). In addition, BrdU^+^ cells were observed in VALT, indicating that local cells undergo proliferation and VALT activation.

Next, we clarified the role of VALT in renal lesion development, as TLT has detrimental effects on the residing organ (21). We found larger VALT as well as more infiltrating immune cells in the kidney, and VALT size was correlated with GL and TIL (**Figure 10**). In addition, there were many immune cells as well as IgM and IgG antibodies at the periphery of VALT invading adjacent tubules and glomerulus. These results indicate that VALT functions as a TLT for the perpetuation of adaptive immune responses by providing a local source of antibody that is generated as a result of local antigen presentation, lymphocyte activation, and maturation in the newly formed structure.

As VALT formation is associated with renal lesions, therapeutics targeting VALT formation could ameliorate renal lesions. This study clearly showed the molecular bases of VALT development, which could be considered as therapeutic targets, including major LCs, mechanical removal of VALT, and administration of immunosuppressive drugs. The first two approaches are not feasible as the administration of antibodies targeting the reduction of LCs might affect lymphoid tissue necessary for body defense. Moreover, VALT surrounds the large vessels, and mechanical removal of VALT affects the vessel. Therefore, mechanical removal of VALT from mouse kidneys as well as human patients suffering from lupus nephritis is not realistic. Therefore, we chose the latter target since VALT developed due to renal inflammatory conditions. Moreover, prophylactic treatment with Dex effectively reduces the incidence of CKD after cardiac surgery (34). Surprisingly, we observed that administration of Dex reduced serum anti-dsDNA antibody and spleen to body weight ratio and almost ablated the VALT with the least expression of LCs (**Figure 11**). Dex has multifaceted effects on different immune cells and can reduce the development of pathogenic lesions directly or indirectly, although this remedy is also accompanied by side effects (15). Therefore, refining the Dex dose, starting time, and duration of administration should be considered before using it as a therapeutic to reduce LN lesions through ablation of VALT.

**Figure 12:**
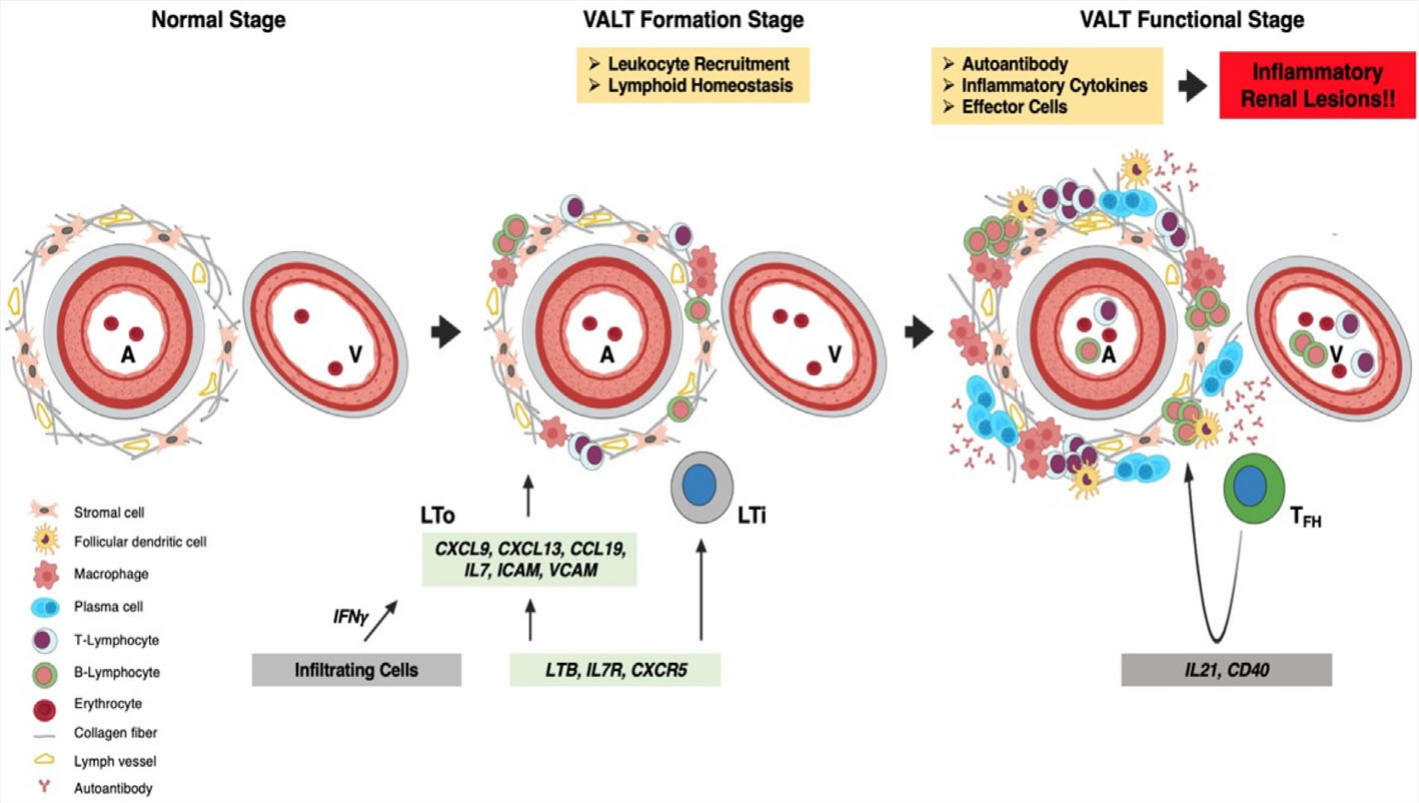
Summary of VALT formation and its effects. The perivascular space contains stromal cells and collagen fibers in the normal kidney. At the VALT formation stage, *Ifng* secreted by infiltrating cells stimulates LTo to secrete LC (*Ccl8, Cxcl9* and *-13*), which attract leukocytes for homing around the perivascular space to form VALT. Interaction between LTi and LTo also increases LC production. The retention of infiltrated cells is maintained by costimulatory molecules (VCAM and ICAM) secreted from LTo. At the functional stage, VALT becomes larger where helper T-cells become follicular T_FH_ cells with progression of inflammation and helps in germinal center formation and activity. VALT functions in the perpetuation of autoantibodies against autoantigens and effector cells, which aggravates inflammatory renal lesions. A, artery; V, vein; LTi, lymphoid tissue inducer; LTo, lymphoid tissue organizer; LC, lymphorganogenic chemokinesand; T_FH_, T follicular helper cell.

In conclusion (**Figure 12**), the perivascular space contains stromal cells and fibers in the normal kidney. At the beginning of LN, *Ifng* from infiltrating cells stimulates stromal cells and fibers to secrete LCs. Recruited LTi cells also stimulate LTo to secrete more LCs to attract leukocytes for homing around the artery to form VALT. With the progression of LN, VALT becomes larger and helper T cells acquire the phenotype of T_FH_ cells, which stimulates B cells for proliferation, affinity maturation, and GC formation. Autoantibodies and effector cells are formed in VALT upon stimulation with autoantigens, which aggravates renal lesions. Moreover, administration of Dex ablates VALT in LN. Therefore, this study revealed the cell types and molecules that governed the formation of VALT in LN, and thereby opening a new window for therapeutic intervention of LN through ablation of VALT.

## Data Availability Statement

The datasets presented in this study can be found in online repositories. The names of the repository/repositories and accession number(s) can be found in the article/**Supplementary Material**.

## Ethics Statement

The animal study was reviewed and approved by Institutional Animal Care and Use Committee of the Faculty of Veterinary Medicine, Hokkaido University, approval No. 16-0124.

## Author Contributions

MM: conceptualization, formal analysis, funding acquisition, investigation, methodology, and roles/writing—original draft. OI: conceptualization, funding acquisition, investigation, and roles/writing—original draft. YE: conceptualization, investigation, roles/writing-original draft. YO: formal analysis, investigation, and methodology. TN: formal analysis, investigation, and methodology. YK: conceptualization, funding acquisition, investigation, supervision, and roles/writing—original draft. All authors contributed to the article and approved the submitted version.

## Funding

This work was supported by JSPS KAKENHI [Grant number 19F19092 and 19K22352]. The funder had no role in study design, execution of experiments, data collection, manuscript preparation, and decision to publish.

## Conflict of Interest

The authors declare that the research was conducted in the absence of any commercial or financial relationships that could be construed as a potential conflict of interest.
