# Supplementary material for: Vasculature-Associated Lymphoid Tissue: A Unique Tertiary Lymphoid Tissue Correlates With Renal Lesions in Lupus Nephritis Mouse Model
Source: Front Immunol. 2020 Dec 15;11:595672. doi: 10.3389/fimmu.2020.595672 (PMC7770167; doi:10.3389/fimmu.2020.595672)
Supplement: Supplementary file 2 [file Table_1.docx]

**Supplementary Table 1:** List of antibody and conditions used for immunohistochemistry/ immunofluorescence

| Parameters | Antigen retrieval | Blocking | Primary antibody | Secondary antibody |
| --- | --- | --- | --- | --- |
| B220 | CB 115°C, 15 min/  TB 115°C, 15 min | 10% NGS/  5% NDS | Rat polyclonal antibodies (Cedarlane, Burlington, Canada) 1:1000 | Goat anti-rat IgG (Caltag Medsystems, Buckingham, UK) 1:100 (Biotinylated)/  Alexa Fluor 488-labeled donkey anti-rat IgG antibodies (1:500; Life Technologies, California, USA) |
| CD3 | TB 115°C, 15 min | 10% NGS/  5% NDS | Rabbit polyclonal antibodies (Nichirei, Tokyo, Japan) 1:200 | Goat anti-rabbit (SABPO kit, Nichirei) 1:100 (Biotinylated)/  Alexa Fluor 546-labeled donkey anti-rabbit IgG antibodies (1:500; Life Technologies) |
| Iba1 | TB 115°C, 15 min/ 0.1% pepsin 37°C, 5 min | 10% NGS/  5% NDS | Rabbit polyclonal antibodies (Wako, Tokyo, Japan) 1: 1200/  Goat polyclonal antibodies (Abcam, Tokyo, Japan) 1: 600 | Goat anti-rabbit (SABPO kit, Nichirei) 1:100 (Biotinylated)/ Donkey anti-goat (Santa Cruz, California, USA) 1:100/  Alexa Fluor 488-labeled donkey anti-rabbit IgG antibodies (1:500; Life Technologies) |
| LYVE 1 | CB 115°C, 15 min | 10% NGS | Rabbit polyclonal antibodies  (Adipogen, San Diego, California, USA) 1: 500 | Goat anti-rabbit (SABPO kit, Nichirei) 1:100 (Biotinylated) |
| PNAd | TB 115°C, 15 min | 10% NGS | Rat polyclonal antibodies  Biolegend  (San Diego, USA) 1: 500 | Goat anti-rat IgG (Caltag Medsystems) 1:100 (Biotinylated) |
| CCL8 | TB 115°C, 15 min | 5% NDS | Goat polyclonal antibodies  (R and D System Minneapolis, USA) 1: 400 | Alexa Fluor 647-labeled donkey anti-goat IgG antibodies (1:500; Life Technologies) |
| CXCL9 | TB 115°C, 15 min | 5% NDS | Goat polyclonal antibodies  (R and D System Minneapolis, USA) 1: 400 | Alexa Fluor 647-labeled donkey anti-goat IgG antibodies (1:500; Life Technologies) |
| CXCL13 | TB 115°C, 15 min | 5% NDS | Goat polyclonal antibodies  (R and D System) 1: 200 | Donkey anti-goat (Santa Cruz) 1:100/  Alexa Fluor 546/647-labeled donkey anti-goat IgG antibodies (1:500; Life Technologies) |
| Vimentin | TB 115°C, 15 min | 5% NDS | Rabbit  Cell signaling (Massachusetts, USA) 1: 1000 | Alexa Fluor 546-labeled donkey anti-rabbit IgG antibodies (1:500; Life Technologies) |
| SMA | CB 115°C, 15 min | 5% NDS | Rabbit polyclonal antibodies  (Abcam)  1: 3000 | Alexa Fluor 488-labeled donkey anti-rabbit IgG antibodies (1:500; Life Technologies) |
| CD21 | CB 115°C, 15 min | 10% NGS | Rabbit polyclonal antibodies  (Abcam) 1: 500 | Goat anti-rabbit (SABPO kit, Nichirei) 1:100 (Biotinylated) |
| BrdU | CB 115°C, 15 min | 10% NGS | Rat monoclonal antibody  (Abcam) 1: 400 | Goat anti-rat IgG (Caltag Medsystems) 1:100 (Biotinylated) |
| MHC II | CB 115°C, 15 min | 5% NDS | Rat monoclonal antibody  (Abcam) 1:300 | Alexa Fluor 488-labeled donkey anti-rat IgG antibodies (1:500; Life Technologies) |
| CD138 | CB 115°C, 15 min | 10% NGS | Rat monoclonal antibody  (Biolegend)  1:300 | Goat anti-rabbit IgG (Caltag Medsystems) 1:100 (Biotinylated) |
| IgM | CB 115°C, 15 min | 10% NGS | Rabbit polyclonal antibodies  (Bethyl laboratories, Montgomery, USA) 1:1500 | Goat anti-rabbit IgG (Caltag Medsystems) 1:100 (Biotinylated) |
| IgG | CB 115°C, 15 min | 10% NGS | Rabbit polyclonal antibodies  (Bethyl laboratories)  1:2000 | Goat anti-rabbit IgG (Caltag Medsystems) 1:100 (Biotinylated) |
| IL1F6 | CB 115°C, 15 min | 5% NDS | Goat monoclonal antibody (R and D System) 1: 400 | Goat anti-donkey IgG (Santa Cruz, California, USA) 1:100 |
| CB: citrate buffer, TB: tris buffer, NGS: normal goat serum and NDS: normal donkey serum | | | | |
