# Supplementary material for: Vasculature-Associated Lymphoid Tissue: A Unique Tertiary Lymphoid Tissue Correlates With Renal Lesions in Lupus Nephritis Mouse Model
Source: Front Immunol. 2020 Dec 15;11:595672. doi: 10.3389/fimmu.2020.595672 (PMC7770167; doi:10.3389/fimmu.2020.595672)
Supplement: Supplementary file 3 [file Table_2.docx]

**Supplementary Table 2**: Details of primers and probes used in this study

| **Gene name** | **Symbol** | **Gene ID** | **Application** | **Company** | Primer sequence (5^’^-3^’^) | **Size** |
| --- | --- | --- | --- | --- | --- | --- |
| Actin, beta | *Actb* | qPCR | qPCR (SYBR) | Eurofins Genomics (Tokyo, Japan) | F: TGTTACCAACTGGGACGACA  R: GGGGTGTTGAAGGTCTCAAA | 165 |
| Chemokine (C-X-C motif) ligand 9 | *Cxcl9* |  | qPCR (SYBR) | Eurofins Genomics (Tokyo, Japan) | F: TGTTACCAACTGGGACGACA  R: GGGGTGTTGAAGGTCTCAAA | 194 |
| Chemokine (C-X-C motif) ligand 13 | *Cxcl13* |  | qPCR (SYBR) | Eurofins Genomics (Tokyo, Japan) | F: TCATCTTCCTGGAGCAGTGTG  R: ATCTAGGCAGGTTTGATCTCCG | 115 |
| Chemokine (C-X-C motif) receptor 5 | *Cxcr5* |  | qPCR (SYBR) | Eurofins Genomics (Tokyo, Japan) | F: ATCCTCGTGCCAAATGGTTACA  R: AGATGATAGTGGCTTCAGGCAG | 133 |
| Interferon gamma | *Ifng* |  | qPCR (SYBR) | Merck (Darmstadt, Germany) | F: ACTACCCACTAACCCTGGACA  R: GGTCCCTCGACTGTAGAGCA | 201 |
| Tumor necrosis factor alpha | *Tnfa* |  | qPCR (SYBR) | Merck (Darmstadt, Germany) | F: CCTTTGGACCCTCTGACTTG  R: TTCCACATCTATGCCACTTGAG | 167 |
| **Gene name** | **Symbol** | **Gene ID** | **Application** | **Company** | Assay ID | **Region** |
| Chemokine (C-C motif) ligand 8 | *Ccr8* | 12776 | ISH | Diagnostic  (Newark, CA, USA)  Advanced Cell | 546211 | 2-498 |
| Chemokine (C-X-C motif) ligand 9 | *Cxcl9* | 17329 | ISH | Diagnostic  (Newark, CA, USA)  Advanced Cell | 489341 | 368-1437 |
| Chemokine (C-X-C motif) ligand 9 | *Cxcl13* | 55985 | ISH | Diagnostic  (Newark, CA, USA)  Advanced Cell | 406311 | 2-1143 |
