# Supplementary material for: Vasculature-Associated Lymphoid Tissue: A Unique Tertiary Lymphoid Tissue Correlates With Renal Lesions in Lupus Nephritis Mouse Model
Source: Front Immunol. 2020 Dec 15;11:595672. doi: 10.3389/fimmu.2020.595672 (PMC7770167; doi:10.3389/fimmu.2020.595672)
Supplement: Supplementary file 4 [file Image_1.pdf]

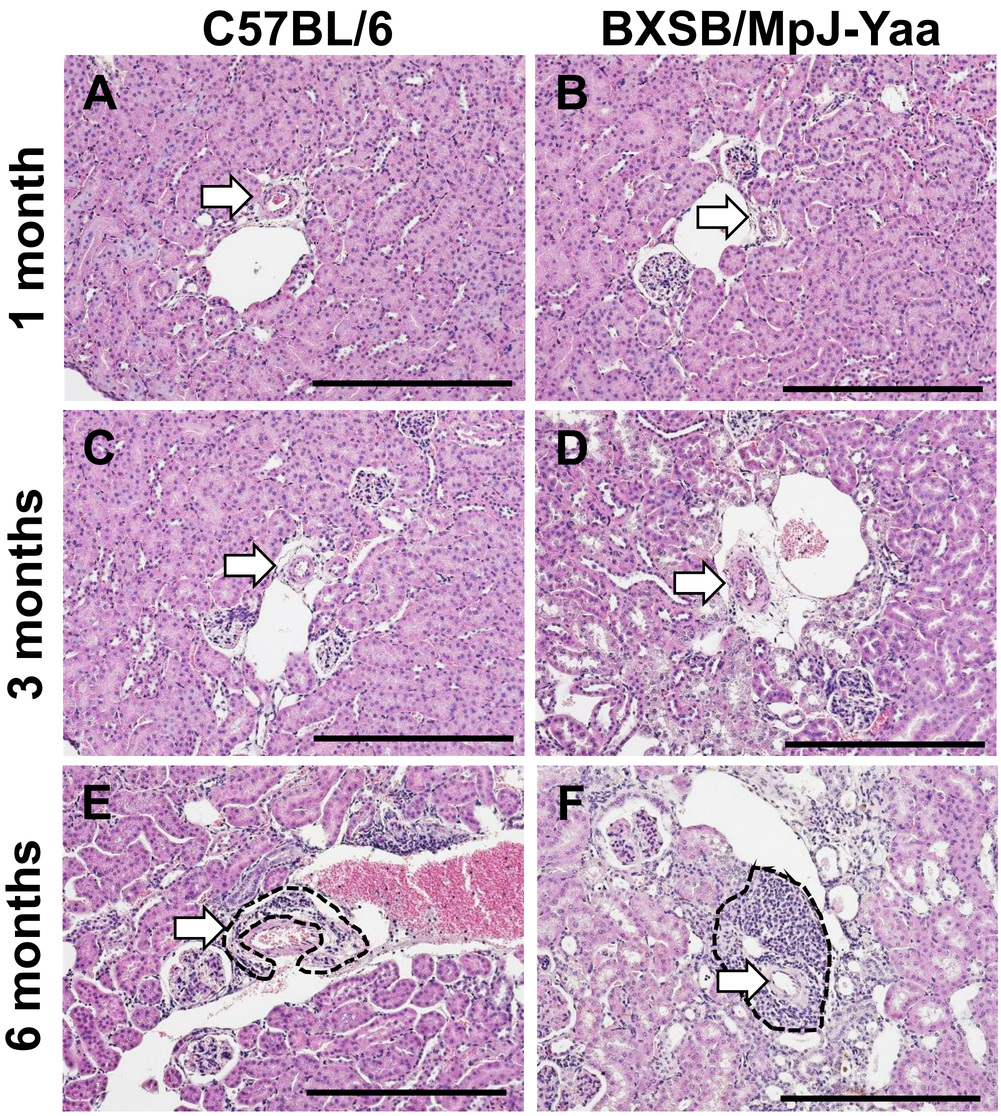

Supplementary Figure 1. Appearance of PCC at different ages of another (BXSB/MpJ-Yaa) autoimmune disease model mice and normal mice kidney.

A-B. Absence of PCC near the blood vessel (arrow) in C57BL/6 and BXSB/MpJ-Yaa mice kidney at 1 month of age.

C-D. Absence of PCC near the blood vessel (arrow) in C57BL/6 and BXSB/MpJ-Yaa mice kidney at 3 months of age.

E-F. Presence of PCC (dashed area) near the blood vessel (arrow) in C57BL/6 and BXSB/MpJ-Yaa mice kidney at 6 months of age (HE stain)

Bars=100 µm. PCC: perivascular cellular cluster, HE: hematoxylin and eosin
