# Supplementary material for: Vasculature-Associated Lymphoid Tissue: A Unique Tertiary Lymphoid Tissue Correlates With Renal Lesions in Lupus Nephritis Mouse Model
Source: Front Immunol. 2020 Dec 15;11:595672. doi: 10.3389/fimmu.2020.595672 (PMC7770167; doi:10.3389/fimmu.2020.595672)
Supplement: Supplementary file 5 [file Image_2.pdf]

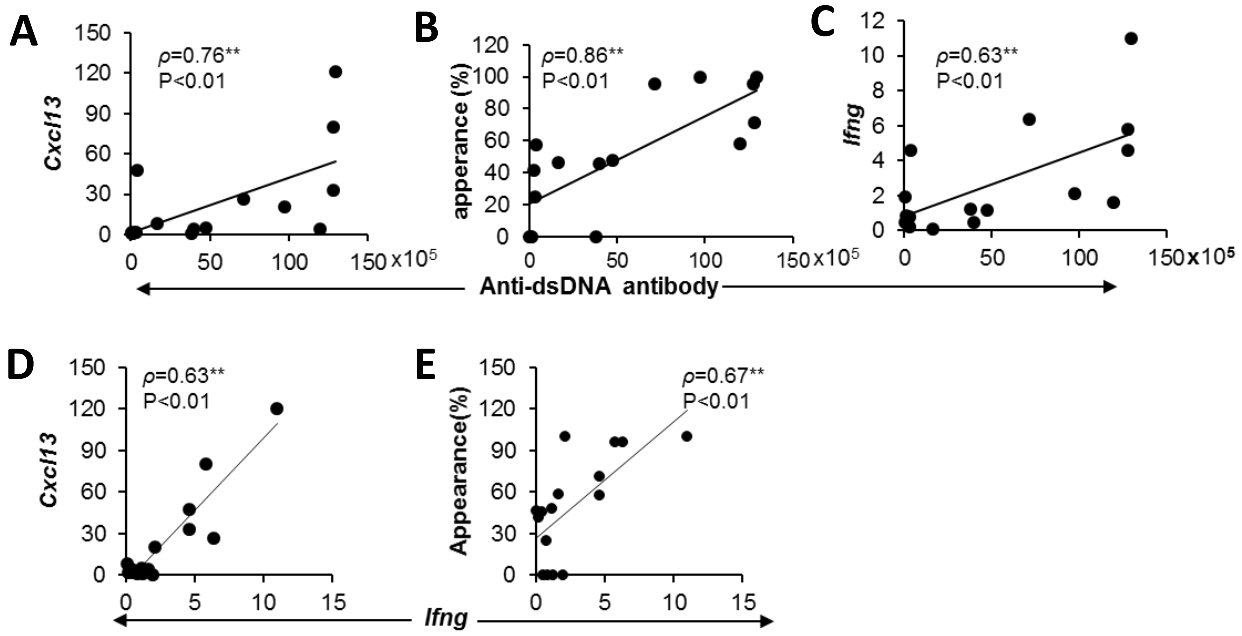

Supplementary Figure 2. Correlation of anti-dsDNA antibody and *Ifng* with PCC appearance and lymphorganogenic chemokines

A-C. Correlation of anti-dsDNA antibody with *Cxcl13* expression, PCC appearance, and *Ifng* expression.
D-E. Correlation of *Ifng* expression with *Cxcl13* expression and PCC appearance
*$P < 0.05$ and **$P < 0.01$, Spearman's rank correlation coefficient, n =16.
Ifng: *interferon gamma* and PCC: perivascular cellular cluster.
