# Supplementary material for: Vasculature-Associated Lymphoid Tissue: A Unique Tertiary Lymphoid Tissue Correlates With Renal Lesions in Lupus Nephritis Mouse Model
Source: Front Immunol. 2020 Dec 15;11:595672. doi: 10.3389/fimmu.2020.595672 (PMC7770167; doi:10.3389/fimmu.2020.595672)
Supplement: Supplementary file 6 [file Image_3.pdf]

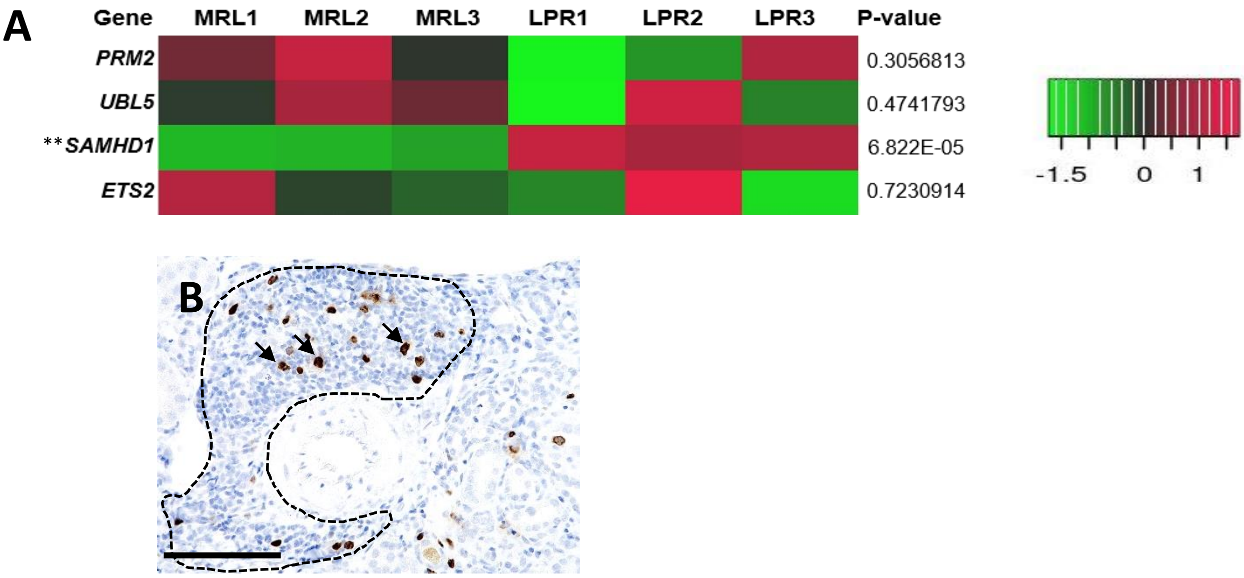

Supplementary Figure 3. Autoantigen in kidney and proliferating cells in the VALT.

A. Gene transcripts for different autoantigens in MRL and LPR mice kidney at 6 months of age. Microarray analysis, significant differences from the control is indicated by * (*$P < 0.05$, **$P < 01$, 2-tailed Student's *t* test). n=3.

B. $BrdU^{+}$ proliferating cells (arrows) in VALT (dashed area) of the kidney of LPR mice at six months of age (IHC)

Bar=100 µm. VALT: vasculature-associated lymphoid tissue and BrdU: bromodeoxyuridine.
